# The Association between Household Socioeconomic Position and Prevalent Tuberculosis in Zambia: A Case-Control Study

**DOI:** 10.1371/journal.pone.0020824

**Published:** 2011-06-17

**Authors:** Delia Boccia, James Hargreaves, Bianca Lucia De Stavola, Katherine Fielding, Ab Schaap, Peter Godfrey-Faussett, Helen Ayles

**Affiliations:** 1 Faculty of Epidemiology and Population Health, Department of Infectious Disease Epidemiology, London School of Hygiene and Tropical Medicine, London, United Kingdom; 2 Faculty of Epidemiology and Population Health, Department of Medical Statistics, London School of Hygiene and Tropical Medicine, London, United Kingdom; 3 ZAMBART Project, Ridgway Campus, University of Zambia, Lusaka, Zambia; 4 Faculty of Infectious and Tropical Diseases, Department of Clinical Research, London School of Hygiene and Tropical Medicine, London, United Kingdom; McGill University, Canada

## Abstract

**Background:**

Although historically tuberculosis (TB) has been associated with poverty, few analytical studies from developing countries have tried to: 1. assess the relative impact of poverty on TB after the emergence of HIV; 2. explore the causal mechanism underlying this association; and 3. estimate how many cases of TB could be prevented by improving household socioeconomic position (SEP).

**Methods and Findings:**

We undertook a case-control study nested within a population-based TB and HIV prevalence survey conducted in 2005–2006 in two Zambian communities. Cases were defined as persons (15+ years of age) culture positive for M. *tuberculosis*. Controls were randomly drawn from the TB-free participants enrolled in the prevalence survey. We developed a composite index of household SEP combining variables accounting for four different domains of household SEP. The analysis of the mediation pathway between household SEP and TB was driven by a pre-defined conceptual framework. Adjusted Population Attributable Fractions (aPAF) were estimated.

Prevalent TB was significantly associated with lower household SEP [aOR = 6.2, 95%CI: 2.0–19.2 and aOR = 3.4, 95%CI: 1.8–7.6 respectively for low and medium household SEP compared to high]. Other risk factors for prevalent TB included having a diet poor in proteins [aOR = 3.1, 95%CI: 1.1–8.7], being HIV positive [aOR = 3.1, 95%CI: 1.7–5.8], not BCG vaccinated [aOR = 7.7, 95%CI: 2.8–20.8], and having a history of migration [aOR = 5.2, 95%CI: 2.7–10.2]. These associations were not confounded by household SEP. The association between household SEP and TB appeared to be mediated by inadequate consumption of protein food. Approximately the same proportion of cases could be attributed to this variable and HIV infection (aPAF = 42% and 36%, respectively).

**Conclusions:**

While the fight against HIV remains central for TB control, interventions addressing low household SEP and, especially food availability, may contribute to strengthen our control efforts.

## Introduction

While the epidemic of tuberculosis (TB) that affected the Northern industrialised countries in 1900s was mainly associated with social, economic, and environmental factors - including increasing population density, urbanisation and poor nutrition [1] - the current epidemic, mainly involves developing countries and appears to result from the complex interplay of *old* socioeconomic determinants and *new* factors, like HIV, the emergence of antimicrobial drug resistance and possibly more virulent strains [2], [3].

Interest in the relative importance of biological and socioeconomic factors in driving this epidemic of “new TB” [4] has been recently stimulated by the work of the Commission on Social Determinants of Health [5] and studies challenging the impact of the current global strategy for the control of TB based on case finding and treatment [6], [7], [8], [9]. In particular, two ecological studies have suggested that broad socioeconomic development, rather than the success of TB control programmes, is the main determinant behind the declining trends of TB observed in many regions of the world [2], [10].

Although socioeconomic factors are likely to remain important key components in the epidemiology of TB in developing countries, it is more challenging to understand *how* inadequate living conditions affect the risk of TB in a given setting, *how* this effect may be mediated by risk factors (especially HIV infection) that are on the causal pathway, and *how* evidence can inform concrete interventions to strengthen the global response to TB. In other words, the question is no longer whether poverty is associated with TB, but why it is so, whether the HIV epidemic may have introduced some discontinuity in our understanding of TB epidemiology and how this should inform TB control policies.

So far these issues have been explored by a surprisingly small number of studies [11], [12], [13], [14], [15], [16], [17], [18], [19], [20], the majority of which suffer from at least two main limitations: 1) a limited conceptual framework to guide the analysis of the causal pathway underlying the association between poverty and TB; and, 2) unclear measurement of socioeconomic position (SEP) at household and/or individual level with little information given on how SEP was defined or operationalised. Further, much of the evidence comes from studies of notified cases of TB. Because such cases have, by definition, been identified by health services they may represent a selected, perhaps wealthier, group of cases, who have overcome the financial barriers that often prevent TB case detection [21], [22]. Such case detection bias can result in the paradoxical impression that TB is more common among wealthier population groups [13] or areas [18].

In 2007 we undertook a case-control study in Zambia to investigate the association between household SEP and prevalent TB in a country affected both by widespread poverty and a severe epidemic of HIV. The study was nested within a large population-based TB and HIV prevalence survey undertaken between 2005 and 2006 in the Lusaka province [23]. The nested design offered a unique platform for the collection of socioeconomic data from prevalent TB cases detected through active-case finding enabling us to minimise the detection bias described above. Furthermore, because prevalent cases are detected while they are still infectious, they should reflect the extent of ongoing transmission of TB in a community. Consequently, the measurement of risk factors in this group, including household SEP, can have important implications for TB control.

The aims of this study were to: 1) quantify the association between household SEP and prevalent TB; 2) explore the potential mechanisms through which household SEP might affect risk of TB disease; and, 3) estimate how many cases of prevalent TB could be prevented by improving household SEP compared to other relevant risk factors.

## Methods

### Study population and study setting

Cases and controls were recruited from among participants in the 2005–2006 population-based TB and HIV prevalence survey conducted in two communities of the Chongwe and Kafue districts of the Lusaka Province, respectively rural and peri-urban [23]. The rural area (∼17,000 inhabitants) is a small farming community where the local economy is driven mainly by agriculture. Other livelihood options include petty vending with only a small proportion of people formally employed. In this area many houses are made of mud bricks and thatched roofs. The peri-urban site (∼11,000 inhabitants) is a small shanty compound surrounded by farms. People living in the peri-urban site are generally poor with few livelihood options. Many of the houses are made of burnt bricks while others of concrete blocks. The TB and HIV prevalence survey recruited individuals aged 15 years and over from randomly sampled households and found the prevalence of culture-confirmed TB to be 870/100,000 [95%CI: 570–1,160/100,000] overall, and 1,200/100,000 [95%CI: 750–1,640/100,000] and 650/100,000 [95%CI: 360–940/100,000] in the peri-urban and rural site respectively [23].

### Study design

Cases and controls were frequency-matched by area of residence and age-group (15–29, 30–44, ≥45). As for the prevalence survey, a case of TB was defined as any having at least one sputum sample culture positive for M. *tuberculosis*. Controls were sputum culture negative for M. *tuberculosis* or any other mycobacteria. Controls were excluded if reporting cough for more than two weeks (to rule out the inclusion of suspected cases of TB) and if they were household members of a recruited case (to avoid overmatching). We dealt with the occurrence of two cases arising from the household by enrolling in the study only the one who was first diagnosed. To be enrolled in the study both cases and controls had to give written informed consent and have both the individual and household-level questionnaires completed.

### Sample size and sampling strategy

Assuming a prevalence of extreme poverty among the controls of 36% [24], we estimated that we needed a total of 100 cases and 300 controls to detect an odds ratio of 2 for TB in individuals with low household SEP with a study power of 80% and 5% significance. Since approximately 100 cases of TB were expected to be found in the prevalence survey (based on an overall sample size of approximately 10,000 individuals and an expected TB prevalence of 1% in the general population) we decided to recruit all TB cases detected in this survey. Controls were randomly drawn from the pool of TB-free participants recruited to the prevalence survey. For each TB case, three controls were then randomly chosen from one of the six matching strata based on age-group and area of residence.

### Microbiological definition of *M. tuberculosis* and number of study participants

**Figure 1:**
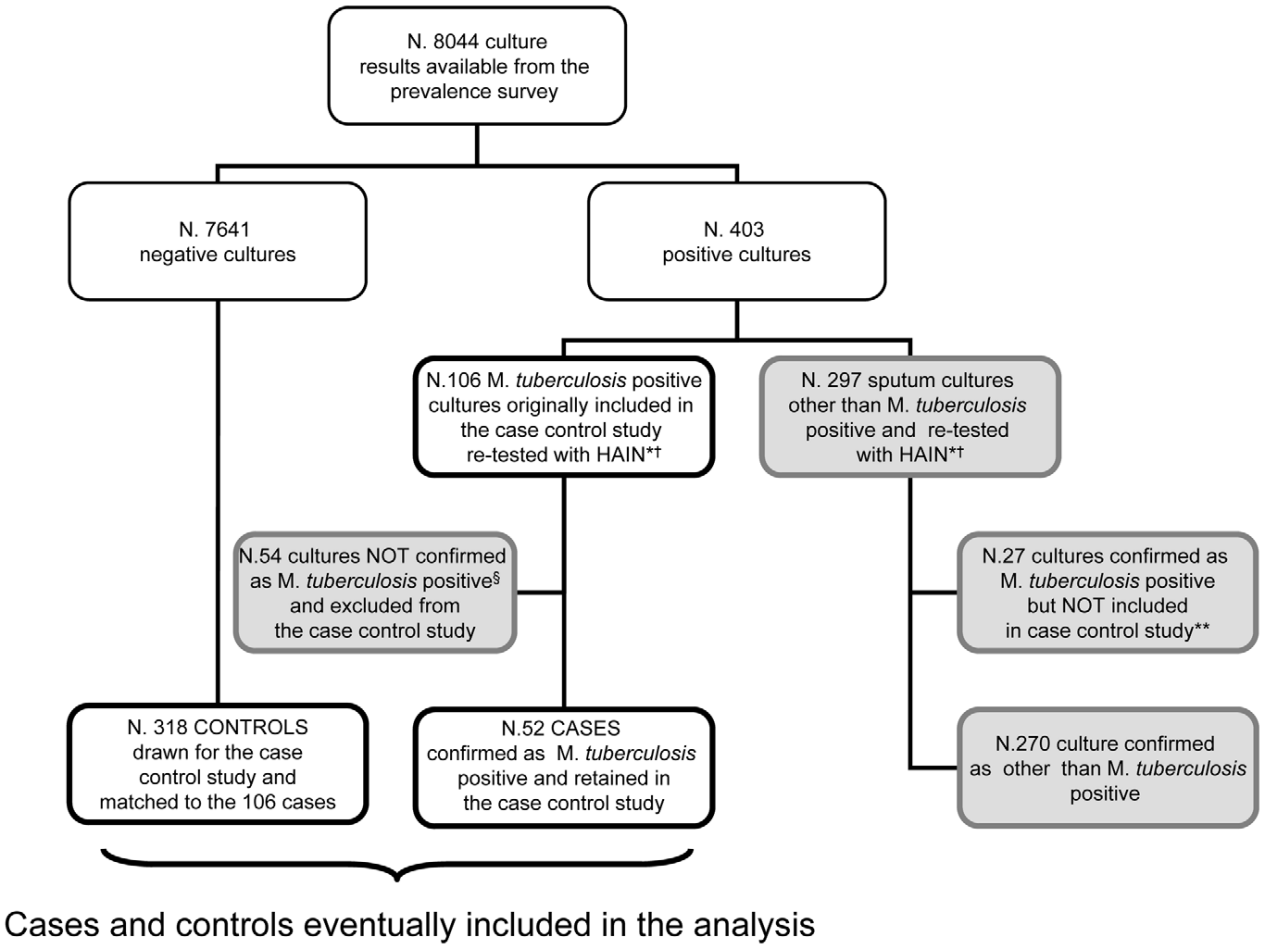
Study design and flowchart of study participants. Grey boxes show the cases of TB that have been not included in the case-control study. *HAIN Life Science, based on acid nucleic amplification technology. ^†^ Samples were re-tested after data on the initial 106 cases and 318 controls had been already collected. ** Overall 79 cases were eventually detected in the prevalence survey. Of them only 52 were included in the present case control study. The remaining 27 were not included because identified after the fieldwork was completed. ^§^The 54 cases excluded were classified as follows: *M. intracellulare* (N. 21), Non Mycobacteria Type 1 (N. 9), *M. scrofulaceum* (N. 3), *M. asiaticum* (N. 2), *M. goodie* (N. 1), *M. gordonae* (N. 1), *M. parafinicum* (N. 1), *M. peregrinum* (N. 1), *M. terrae* II (N. 1). The remaining 14 strains were classified as unidentified Mycobacteria species.

Initially 106 out of the 403 cultures showing evidence of growth were identified as M. *tuberculosis* positive through the niacin accumulation test and/or identified by spoligotyping. Based on the study protocol, all these cases were included in the case-control study together with 318 controls (Figure 1). Two months after the completion of data collection, all the 403 cultures were re-tested with the Genotype Mycobacteria CM Assay (HAIN test. Life Science), a nucleic acid amplification-based technology known to be highly specific for M. *tuberculosis* [25]. Only 52 of the 106 cases initially identified were confirmed to be M. *tuberculosis* (Figure 1).

To ensure consistency with the microbiological case definition adopted in the prevalence survey and to avoid any dilution effect following from the inclusion of cases of non-tuberculous mycobacteria, we restricted the data analysis only to those 52 cases of M. *tuberculosis*, as defined by the Genotype CM assay. All the 318 controls were retained in the data analysis to maximise study power.

### Conceptual framework

**Figure 2:**
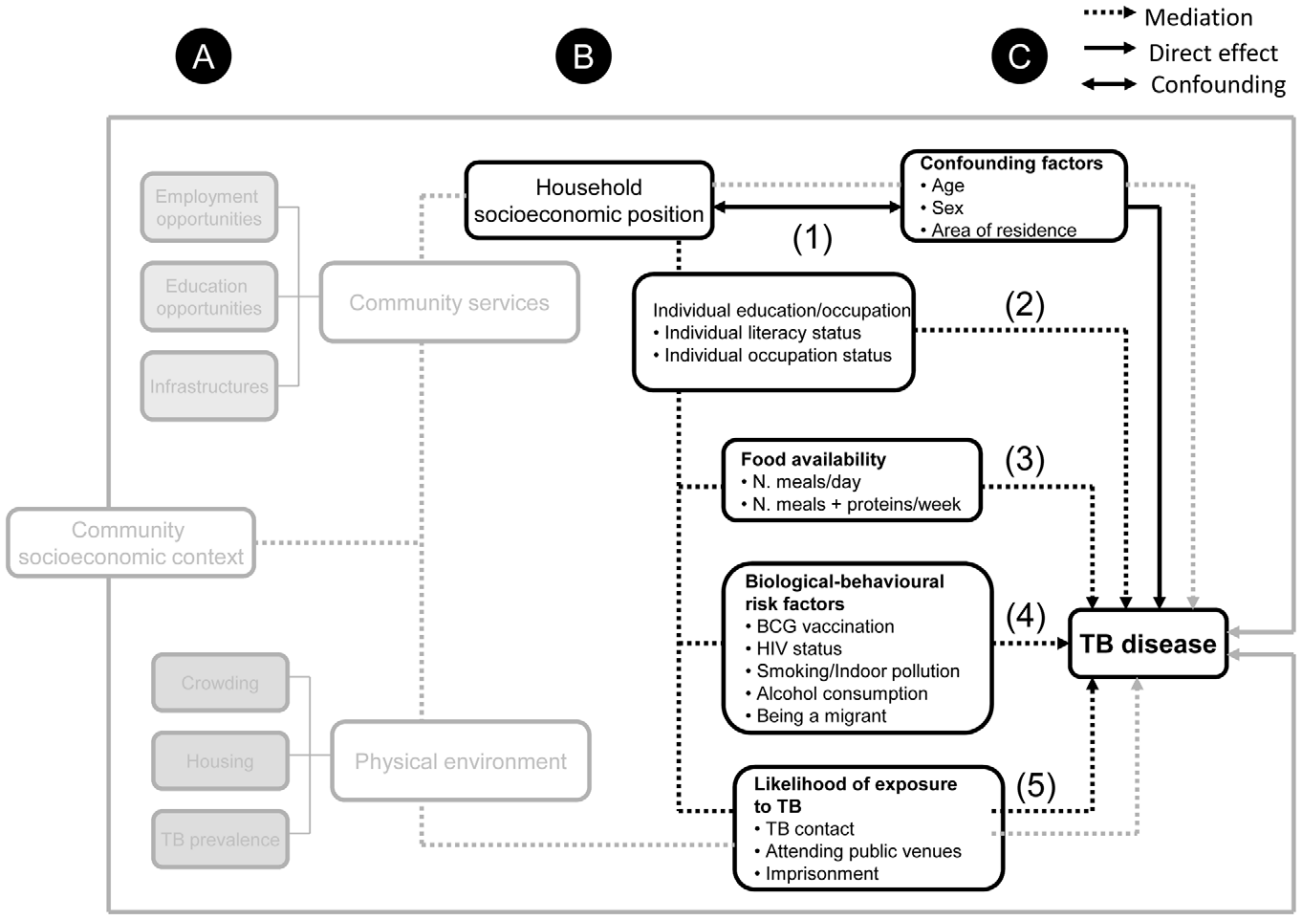
The study conceptual framework. The grey lines and boxes show the postulated association between community socioeconomic position and TB disease. This analysis is not part of the case control study and it is not discussed in this paper. For the assessment of the association between household socioeconomic position and TB disease we took into account potential confounders (indicated by the ↔ line) and four mediation pathways (indicated by the dotted arrow). The unmediated association between household socioeconomic position and TB disease is indicated by the continuous arrow . Number in brackets show the four multivariable models run for the analysis and the independent variables included. Model (1): Household SEP minimally adjusted for sex, age group and area of residence Model (2): as model 1 plus education/occupation-related variables Model (3): as model 2 plus food intake-related variables Model (4): as model 2 plus biological-behavioural risk factors for TB Model (5): as model 2 plus TB exposure-related variables.

Data collection and analysis were driven by a pre-defined conceptual framework reflecting the study hypotheses (Figure 2). In particular, we hypothesised that TB disease results from the interplay of risk factors at three different levels: A) the community; B) the household; and, C) the individual. The standards of living in a community may shape household SEP, which in turn might influence individual *opportunities* (in terms of education, occupation, nutrition, housing quality and social interaction) and the health-related *choices* (such as smoking, alcohol consumption, HIV exposure, BCG vaccination). For the case-control study we focused separately on the household and individual levels and hypothesised that household SEP might affect the likelihood of TB disease by reducing the education and occupation opportunity of the individuals. This in turn may lead to TB either by 1) limiting individuals' food availability; 2) increasing the likelihood of exposure to biological-behavioural risk factors, including HIV infection; and 3) increasing the chance of contact with other TB cases (Figure 2).

With respect to the conceptual framework above, three aspects are worth noticing: 1) although likely to be correlated, household SEP and individual occupation and education level should be considered as independent as they belong to two different causation levels; 2) these factors have been selected after an extensive review of the literature on the most common risk factors for TB prevalence and their likely association with household SEP. Finally, 3) in this study we did not consider these risk factors as potential confounders of the association between household SEP and TB; rather we explored whether our data supported the hypothesis that they might sit on the causal pathway and mediate any association seen between household SEP and TB.

### Ethical approval

Ethical approval was obtained both from the Ethics Committee of the London School of Hygiene and Tropical medicine and the University of Zambia. Informed written consent was obtained from all the study participants.

### Data collection and operationalisation

Household SEP and individual-level data were collected through two separate structured questionnaires. People originally recruited for the prevalence survey were interviewed a second time for the present study. Data were collected over 12 months (March 2006–March 2007), double entered and checked using Epi-Info 6.4.

#### Household socioeconomic position

Household SEP was defined as the complex of social and economic factors that influences what position(s) individuals and groups hold within the structure of the society [26]. We considered four domains we felt relevant to socioeconomic position among the study population: (a) household human resources; (b) household food availability and vulnerability; (c) housing quality and asset ownership; (d) access to community services and infrastructure. Data on variables relevant to these domains were collected from the head of the household if he/she was available or from another household member able to answer the questions. Data were collected at home from field workers that already knew the households; however, most of the household SEP measurement was based on objective observations.

An overall index of relative household SEP was derived by performing a principal component analysis (PCA) [27]. After a screening process guided by the literature [28], [29], only 11 of the 34 initially available socioeconomic variables were included in PCA. The analysis was initially restricted to the controls data, then the weights of the the first principal component were applied to all study participants. These weights were used to create a composite household SEP score for each household enrolled in the study. In addition to this overall index of relative household SEP, four further indices were created using the same data reduction strategy, one for each of the four SEP domains described above. Each of these five indices was categorised into thirds using the 33% and 67% cut-offs to generate tertile groups corresponding to Low, Medium and High household SEP. From the household SEP conceptualisation and operationalisation, it follows that in these communities having higher SEP means having better food security, higher human capital, better housing and assets ownership and living closer to the community's services and infrastructures.

#### Individual level variables

Household SEP data were complemented by data on individual-level mediating factors identified in the conceptual framework (Figure 2). HIV status was based on laboratory results obtained during the prevalence survey [23]. BCG vaccination was assessed by examination of a visible BCG scar on the arms of the recruited cases and controls. Individual-level data were collected directly from cases/controls.

### Data analysis

Data analysis included four components:

#### Description of the general population socioeconomic profile

Control households were characterised by socioeconomic variables and household SEP ranking. All household SEP indices were analysed both as categorical and continuous variables. We used chi square and Mann-Whitney tests to compare respectively the proportion of households classified as “low” SEP and the median household SEP between peri-urban and rural households.

#### The assessment of risk factors for prevalent TB

We first assessed the association between prevalent TB and household SEP, household SEP domains and other relevant risk factors. In all three cases, conditional logistic regression was used to estimate adjusted odds ratios (aORs) and 95% confidence intervals, using the six frequency matched strata of age group and area. This approach was taken because we could not exclude that the matching between cases and controls was to some degree maintained even after the loss of cases. Further, with the small number of cases in this study, reducing the number of parameters in adjusted models was felt to be advantageous. In this first analysis household SEP and all the household SEP indices were treated as tertile variables. All associations were always minimally adjusted for sex and – via conditional logistic regression - for age group and area of residence. In the analysis of non-SEP TB risk factors we further adjusted for the potential confounding effect of household SEP. The likelihood ratio test was used to assess association of factors with being a case as well as interaction between factors and linear trend for ordered categorical variables.

#### The analysis of mediation pathway

After the minimally adjusted analysis we undertook the mediation pathway analysis. In this case household SEP was recoded as a binary variable (Low/High household SEP using the median as cut point) to further reduce of the number of parameters to be estimated. Overall five different models were fitted, again using conditional logistic regression, starting with Model 1 which included only household SEP, sex and age group and area of residence.

Potential mediating factors were subsequently included in a hierarchical fashion following the order outlined in Figure 2 (Model 2–5). Mediation was assessed by including blocks of variables (i.e. education/occupation, food availability/vulnerability, biological/behavioural and TB-exposure related variables) except for HIV infection whose potential mediating effect was studied in combination with the other biological and behavioural-related variables and on its own. Evidence of mediation was detected by comparing the OR for household SEP from Model 1 (*before* mediation) with OR from the remaining models (*after* mediation). Any reduction in the magnitude of the OR from Model 1 was interpreted as evidence of mediation, i.e. that variables added in a given model were explaining part of the association between household SEP and TB prevalence. Each mediator-adjusted OR for household SEP was interpreted as the part of the effect of household SEP that was not mediated by the risk factors included in the model [30].

#### The estimate of the Population Attributable Fraction (PAF)

The PAF for TB risk factors was based on a new multivariable model (Model 6) that included all variables significantly associated with prevalent TB [31]. Only variables found to be significant at 5% level when reciprocally adjusted were retained in the model and used to compute PAF. Each PAF was interpreted as the proportion of prevalent cases of TB that could be attributed to each risk factor, after controlling for each other and for known confounders.

All data analyses were performed with STATA 9.0 (Stata Corporation, College Station, TX).

## Results

Overall 52 cases and 318 controls were included in the analysis. 46.1% of cases were female vs 55.7% among controls, but this difference was not statistically significant (P = 0.3). 89 out of the 318 selected controls had to be replaced mainly because they had moved somewhere else (respectively 47.7% in the urban area and 82.2% in the rural area). Only a small percentage of controls was replaced because they declined participation (18.2% and 2.2% respectively in the urban and rural area). When the controls included in the analysis (N. 318) and the excluded ones (N. 89) were compared, they did not appear to be significantly different for any of the socio-demographic variables considered.

Cases had a mean age of 36 years, with more than half of them concentrated in the age group 30–44 years. Because of the initial frequency-matching design, this age distribution was also reflected among the controls.

### Socioeconomic profile of the sampled population

The first principal component showed an Eigen value of 3.6 and accounted for 33% of the total variance of the variables included in the PCA. Among controls, the median SEP score was 0.1 (range −3.9 to +3.2). Rural households were disproportionately more likely than peri-urban households to be classified as low SEP (95.4% vs 4.8% respectively, P<0.001), whereas the majority of the peri-urban households occupied the top tertile (76.3% compared to 23.7% of rural households, P<0.001). While relatively better off, peri-urban households were still poor: almost 43% of urban residents reported meals containing proteins less then twice a week, while 26% reported not having had enough to eat for more than three months over the 12 months before the interview. Even in the peri-urban area, only 40.0% of the population had access to electricity and private piped water.

### Risk factors for prevalent tuberculosis

Low household SEP had a strong effect on the odds of being a case of prevalent TB (OR = 6.2, 95%CI: 2.0–19.2 and OR = 3.4, 95%CI: 1.5–7.6 respectively for the low and medium SEP group compared to the baseline; overall P<0.001) (Table 1). The association between household SEP and TB in the peri-urban area appeared to be stronger than in the rural area, though there was little evidence of interaction (test for interaction, P = 0.50). For this reason rural and peri-urban households were pooled in later analyses.

**Table 1:** Household SEP and prevalent TB: results from the minimally adjusted analysis, overall and by area of residence.

	Overall^a^				Peri-urban area^b^				Rural area^c^			
	Cases	Controls			Cases	Controls			Cases	Controls		
	N (col. %)	N. (col. %)	Adj.*OR (95%CI)	P value	N. (col. %)	N. (col. %)	Adj.*OR (95%CI)	P value	N. (col. %)	N. (col. %)	Adj.*OR (95%CI)	P value
Household SEP												
Low	18 (34.6)	105 (33.0)	6.2 (2.0–19.2)	<0.001	2 (6.2)	5 (3.4)	4.7 (0.7–29.4)	0.03	16 (84.2)	100 (58.5)	3.9(0.5–31.1)	0.08
Medium	24 (46.1)	99 (31.1)	3.4 (1.5–7.6)		22 (66.7)	55 (37.4)	3.7(1.6–8.8)		2(10.5)	44 (25.7)	1.2 (0.1–13.9)	
High	10 (19.2)	114(35.0)	1.0		9 (27.3)	87 (59.2)	1.0		1 (5.3)	27 (15.8)	1.0	

a Overall = N. 52 cases; N = 318 controls.

b Peri-urban area = N. 33 cases; N. 147 Controls;

c Rural area = N.cases 19; N.171 controls.

Col = column; OR = odds ratio; CI = confidence interval;

*Adj = adjusted for sex, age group and area of residence (minimal adjustment).

Test for interaction, P = 0.5.

**Figure 3:**
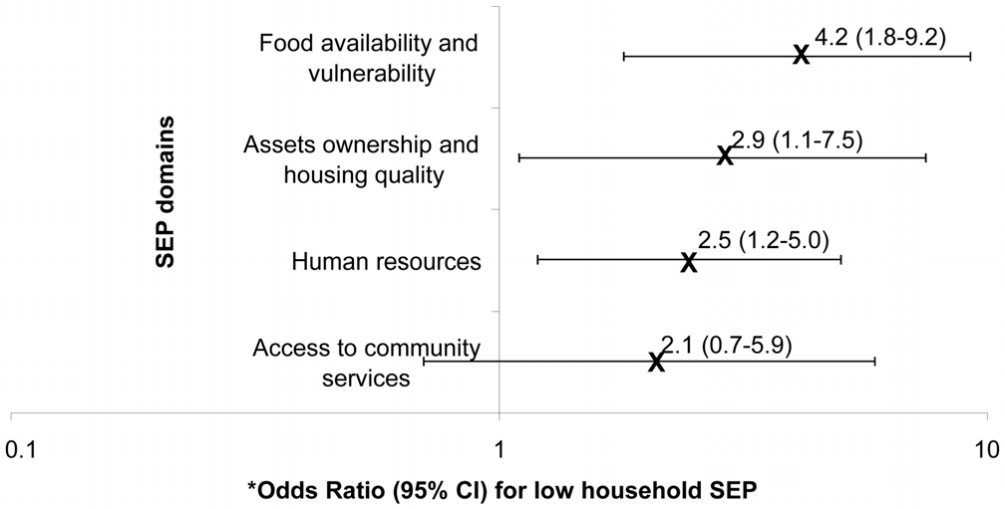
The association between household SEP domains and prevalent TB. Odd Ratios for low household SEP category and 95% Confidence intervals are plotted on a log scale. *After minimally adjusting for sex, age group and area of residence.

With the exception of access to community services, all the indices of SEP domains showed evidence of an association with prevalent TB (Figure 3). The strongest association was observed for the food availability and vulnerability-related domain followed respectively by the assets ownership and housing quality domain and the human resources one. However, it is difficult to rank these domains in terms of association with prevalent TB because the confidence intervals of their respective odds ratios partially overlap.

No *socio-demographic* factor was associated with prevalent TB (Table 2). When looking at *food availability* variables, there was some evidence that having ≤2 meals per day was associated with prevalent TB (OR = 1.8, 95%CI: 0.9–3.4), but this association did not reach statistical significance. People reporting 1 or less meals per week containing proteins had nearly twice the odds to be a case of prevalent TB compared to the reference group. The association between the food-related variables and prevalent TB was attenuated after adjustment for household SEP (Table 2). Among the *biological-behavioural risk factors*, prevalent TB was associated with the lack of BCG vaccination, HIV infection, alcohol consumption and migration, with ORs ranging between 2 and 7.7. These associations persisted after adjusting for household SEP, except for alcohol abuse for which we found evidence of confounding from household SEP. No evidence of association was found between smoking or indoor pollution and being a case of prevalent TB (Table 2). Finally, among the *TB exposure* related variables, having been in contact with anyone with TB in the 12 months prior the interview showed a strong association with TB (Table 2). This association persisted after controlling for household SEP.

**Table 2:** Individual-level risk factors for prevalent TB.

	N. Cases (col. %)	N. Controls (col. %)	Adj OR(a)(95% CI)	P value	Adj OR(b)(95% CI)	P value
Socio-demographic factors						
Female	24 (46.1)	177 (55.6)	0.7 (0.4–1.3)	0.3	0.8 (0.4–1.4)	0.4
Being illiterate	5 (9.6)	46 (14.5)	1.3 (0.5–3.8)	0.6	1.4 (0.5–3.8)	0.7
Highest educational grade achieved						
1–4	4 (8.2)	39 (13.4)	1.0	0.4	1.0	0.2
5–7	24 (49.0)	111 (37.1)	1.8 (0.6–5.8)		2.1 (0.6–6.6)	
8–9	15 (30.1)	8 (27.4)	1.6 (0.5–5.4)		1.8 (0.5–6.3)	
10–12	3 (6.1)	56 (18.7)	0.4 (0.1–2.2)		0.7 (0.2–3.9)	
College	3 (6.1)	11 (3.7)	1.9 (0.4–10.0)		3.6 (0.6–21.2)	
Employment status						
Employed	16 (31.0)	89 (28.0)	1.0	0.6	1.0	0.6
Self-employed	17 (32.7)	103 (32.4)	1.0 (0.4–2.0)		0.7 (0.3–1.5)	
Unemployed/other	19 (36.5)	126 (39.6)	1.2 (0.6–2.6)		0.9 (0.4–2.0)	
Food availability factors						
≤2 meals/day	23 (44.2)	97 (30.5)	1.8 (0.9–3.4)	0.5	1.3 (0.6–2.6)	0.5
N. meals with proteins/week						
>2	6 (11.5)	80 (25.2)	1.0	0.001	1.0	0.07
2	6 (11.5)	61 (19.2)	1.3 (0.4–4.3)		1.1 (0.3–3.6)	
1	24 (46.1)	94 (29.6)	3.8 (1.4–10.0)		2.7 (1.0–7.4)	
0	16 (30.8)	83 (26.1)	3.1 (1.1–8.7)		2.0 (0.6–6.0)	
Biological - behavioural factors						
Not having BCG (N.6)*	9 (18.7)	10 (3.2)	7.7 (2.8–20.8)	<0.001	6.1 (2.2–17.1)	0.001
Being HIV positive (N.4)*	29 (55.8)	89 (28.3)	3.1 (1.7–5.8)	0.001	3.2 (1.5–7.2)	<0.001
Alcohol abuse†	23 (44.2)	88 (27.7)	1.8 (1.0–3.4)	0.05	1.6 (0.9–3.1)	0.1
Cigarette smoking	10 (19.2)	44 (13.8)	1.5 (0.7–3.5)	0.3	1.5 (0.6–3.3)	0.4
Indoor air pollution	44 (84.6)	249 (79.0)	1.4 (0.6–3.1)	0.4	1.2 (0.5–2.7)	0.7
Migration‡	21 (40.4)	44 (13.8)	5.2 (2.7–10.2)	<0.001	5.3 (2.7–10.7)	<0.001
TB exposure factors						
Known contact with TB case (N. 48)*	19 (38.8)	66 (24.2)	2.8 (1.3–5.6)	0.005	2.4 (1.2–5.0)	0.01
Attending:						
Video clubs (N.1)*	3 (5.8)	26 (8.2)	1.0 (0.3–3.6)	>0.9	0.9 (0.2–3.2)	0.8
Bars (N.2)*	12 (23.5)	66 (20.8)	1.2 (0.6–2.4)	0.7	1.1 (0.6–2.3)	0.8
Hairdressing shops (N.1)*	35 (67.3)	200 (63.1)	1.1 (0.6–2.1)	0.8	1.1 (0.6–2.2)	0.8

Col = column; OR = odds ratio; CI = confidence interval.

(a) Adjusted for sex, age group and area of residence (minimal adjustment).

(b) Adjusted for sex, age group, area of residence (minimal adjustment) and household SEP (in tertile format).

*Missing values.

†Drinking more than three drinks containing alcohol every time he/she drinks;

‡Migration was defined as having lived anywhere else for more than six months in the five years before the interview.

### The mediation pathway

Model 1 showed that lower household SEP was associated with TB when household SEP was analysed as binary variable (OR of low household SEP versus high SEP = 2.6, 95%CI: 1.2–5.5 after controlling for age group, sex and area of residence) (Table 3). Only the inclusion of food intake-related variables (Model 3) led to a major reduction in the magnitude of the household SEP's OR suggesting some evidence that the association between SEP and TB was mediated by this block of variables (OR after adjustment = 1.8, 95% CI: 0.7–4.2 P = 0.2) (Table 3). The inclusion of TB exposure-related variables (Model 5) also caused a reduction in the OR of SEP. However, the reduction was less prominent and household SEP was still significantly associated with prevalent TB after adjustment (OR = 2.3, 95%CI: 1.1–5.2, P = 0.04) (Table 3). The size of OR for household SEP remained virtually unchanged after the inclusion of education/occupation variables (Model 2) and the biological/behavioural related variables (Model 4), suggesting no evidence for mediation. This was observed also when HIV infection status was the only added variable to the model with household SEP (Table 3).

**Table 3:** Household SEP and TB: results of the mediation pathway analysis.

	SEP index adjusted OR (95% CI)	P-value
**Model 1** *		
High household SEP**	1.0	0.01
Low	2.7 (1.2–5.9)	
**Model 2** * **– Education/Occupation**		
High	1.0	0.01
Low	2.7 (1.2–6.1)	
**Model 3** * **– Food availability/vulnerability**		
High	1.0	0.2
Low	1.8 (0.7–4.2)	
**Model 4** * **– Biological/behavioural risk factors**		
High	1.0	0.04
Low	2.6 (1.1–6.3)	
**Model 5** * **– TB exposure**		
High	1.0	0.04
Low	2.3 (1.1–5.2)	

*Analysis restricted to 45 Cases and 268 Controls to make the models comparable.

**Household SEP treated as binary variable in the mediation pathway analysis.

OR = odds ratio; CI = confidence interval.

Model 1: household SEP adjusted for sex, age group and area of residence (minimally adjusted).

Model 2: household SEP adjusted for sex, age group, area of residence, education and occupation.

Model 3: as in model 2 plus food intake related variables.

Model 4: as in model 2 plus behavioural risk related variables.

Model 5: as in model 2 including TB exposure related variables.

### The population attributable fraction

The variables used to build the model for the computation of PAFs (Model 6) included sex, age group, area of residence, household SEP and all the risk factors found to be significantly associated with TB as indicated in Table 2. Because household SEP and TB contact were no longer significantly associated with prevalent TB in this multivariable model, they were excluded to gain parsimony (Table 4). This simpler model showed that in this population the highest proportion of cases of prevalent TB could be attributed to HIV infection and to the weekly number of meals containing proteins, with adjusted PAFs equal to 35.8% (95%CI: 15.3–51.4) and 41.7% (3.0–64.6), respectively. In contrast, despite the observed strong effect, not having BCG vaccination exhibited the smallest PAF (11.2%, 95%CI: 2.0–19.0) because of the low frequency of unvaccinated people in this population. Finally, 23.4% (95%CI: 9.0–35.6) of the cases in this setting could be attributed to migration.

**Table 4:** Population attributable fraction.

	% Exposed among cases	Adj. OR(95% CI)	P-value	Adj. PAF	95% CI
Model 6*					
Weekly N. meals + proteins					
0	30.8	2.7 (0.9–8.5)	0.02	41.7%	3.0–64.6
1	46.2	3.2 (1.2–9.1)			
2	11.5	0.9 (0.2–3.4)			
>2	11.5	1			
Not having BCG	18.8	5.8 (1.8–18.6)	0.03	11.2%	2.0–19.0
Being HIV positive	55.8	3.9 (1.9–7.0)	<0.001	35.8%	15.3–51.4
Migration	40.4	4.2 (1.9–9.3)	<0.001	23.4%	9.0–35.6

OR = odds ratio; CI = confidence interval; Adj = adjusted.

*Model 6: It includes sex, age group, area of residence all the variables significantly associated with prevalent TB, including household SEP, weekly number of meals containing proteins, lack of BCG, HIV status, migration and TB contact. In the multivariable analysis household SEP and TB contact were no longer significantly associated with the outcome and were therefore excluded from the model. The final model included only the variables shown in the table.

## Discussion

This study showed a strong association between household SEP and prevalent TB among a Zambian population. We also found evidence that household food availability and vulnerability was the household SEP domain driving this association. After controlling for household SEP, people were significantly more at risk for prevalent TB if they reported a diet poor in proteins, were HIV positive, not BCG vaccinated, and had spent at least six months away from their communities in the five years before the interview.

When household SEP and individual-level risk factors where combined into a model driven by the conceptual framework it appeared that the association between household SEP and TB disease was at least partially mediated by inadequate consumption of meals containing proteins. The PAF estimates suggest that 42% of the cases could be explained by inadequate consumption of proteins, followed by HIV (PAF = 36%).

This study is one of the few investigations aiming to address the association between household SEP and TB cases detected within a prevalence survey [17], [32], [33]. Prevalent cases of TB are likely to represent the end point of several processes (such as the risk of TB infection, the risk of TB progression, the risk of inadequate health seeking behaviour, poor treatment and compliance), each of which may be affected by household SEP. As a result, the household SEP effect we observed is likely to reflect this combined effect on each of these stages. This is a strength of this study, but also a limitation as it makes the understanding of household SEP's role less straightforward. We may have focused on incident cases (i.e. individuals who develop TB), but there is no easy way to measure TB incidence in a community. The proxy most commonly used for incident cases is notified cases, but this would have biased the effect of household SEP as lower SEP people may face greater barriers to use the health services and so be missed. Furthermore, prevalent cases of TB are more likely to reflect ongoing transmission in a community, so that focusing on prevalent TB cases seems more appropriate than using notified cases to better inform policy for TB control.

It may be argued that in such small, poor communities living conditions are relatively too homogenous to be able to identify differences between low and high SEP households. This is likely to be true; however, we found evidence of a socioeconomic gradient that - although small – was sufficient to detect a difference in the distribution of TB across socioeconomic strata. In other words, it is possible that even if high SEP households in these communities are likely to be still poor, there is something in their being “relatively wealthier” that makes them apparently less vulnerable to TB disease.

The strong association between low household SEP and TB disease is in contrast with our findings in a related study from the same population where we found that higher, rather than lower, household SEP was associated with greater odds of TB infection [34]. Because the two studies involved the same population and the same strategy was adopted for the measurement and analysis of the effect of household SEP, the opposite effect of household SEP identified in the two studies is unlikely to be due to methodological differences across the two studies. A possible explanation for these apparently contrasting results could be that while prevalent TB cases infect a mixed pool of people with different socioeconomic position, those with higher household SEP are more likely to be infected because of the frequency and dynamics of human interaction conferred by their higher SEP [34]. In the pool of TB infected, however, the poor will be those more likely to progress to TB disease.

This study was explicitly designed to overcome some of the main limitations of studies addressing the role of household SEP in the epidemiology of prevalent TB; however, at least three main weaknesses remain. First, because of the cross-sectional design of the study we could not accurately measure the effect of household SEP *before* the onset of TB disease. This temporality issue is complicated by the fact that TB disease is known to have a strong impoverishing effect on individuals and households [18], [35]. Consequently, it is unclear whether the living standards of the households of TB cases had changed since the onset of disease. We tried to minimise this reverse causation bias by choosing an SEP measurement strategy based on assets. Assets are considered to be “slow moving” [36]; in other words, even important changes in the household SEP may leave these assets relatively unchanged in the medium-term [37]. Another source of reverse causality may be the fact that some of the investigated risk factors (such as being incarcerated, a migrant or having alcohol abuse problems) can also influence household SEP. As above, the cross-sectoral nature of the study does not permit to disentangle the direction of causality. However, because we measured household SEP in terms of assets (and therefore capturing long-term wealth), we would be more inclined to believe that household SEP tends to influence these risk factors more than being influenced by them.

The sample size is the second biggest limitation of this study, but was beyond our control. TB is a relatively rare disease and even high TB burden countries rarely present with TB prevalence higher than 1,200/100,000 population [21]. Consequently, studies based on prevalent cases of TB often rely on a small number of cases, which is often no bigger than a few hundreds even in prevalence surveys involving large study population [38]. Having said that, the measure of associations we found were sufficiently large and thus unlikely to be explained by chance.

It may be also argued that because controls were originally age-matched to TB cases, the controls sampled may have been older than the general population and this may have introduced some bias in the ascertainment of household SEP. Nonetheless, this seems to be unlikely as in this study age and household SEP did not appear to be correlated (data not shown). Further, even if this was the case, we would expect that the association between SEP and prevalent TB to be biased towards one.

Another limitation is that we accounted only for a relatively small number of a priori confounding factors, namely age, area of residence and sex. We thus cannot exclude that residual confounding factors may have an impact on our results. Excluding important confounders between our selected mediators and the outcome, TB disease, may also lead to biased estimates of the effect of SEP on TB [39].

Furthermore, there is the chance that measurement or misclassification error may have affected our results. For example, the data on proteins consumption were based on participants self-reports rather than proper food diaries, anthropometric measurements, or nutritional validated questionnaires. Also the measurement of household SEP may have been subject to measurement errors mainly due to the assets included in the index and the weighting strategy that was derived from them [40]. It could also be that TB-affected households may have reported a lower SEP than their actual one because of awareness of the impact TB on poverty or – as opposite - that TB-affected families may have pretended to belong to higher SEP (because afraid of the stigma associated with TB). We cannot say which bias is more likely to occur in these communities; however, we do know that at least half of the operational variables used for the measurement of household SEP were based on the objective observation of the interviewers. Hopefully, using information drawn from objective observations should mitigate any systematic biases in the reporting of household SEP.

Finally, although our conceptual framework was derived from an extensive review of the literature on TB risk factors and their links with SEP, it only examines some of the possible mechanisms underlying the role of household SEP on TB prevalence. Alternative mechanisms should be investigated using – as we did here- pre-specified mediation steps to better exclude potentially spurious observations [41].

### The role of household SEP and HIV on prevalent TB in Zambia

This study added two observations to the evidence of a socioeconomic gradient in the distribution of prevalent TB in the study population. First we showed that household food availability and vulnerability was the household SEP domain mainly driving this gradient. Our analysis suggests that this domain of SEP was more strongly associated with TB than education, housing quality or access to community services. This adds to our understanding of the social epidemiology of TB in Zambia.

Second, the association between household SEP and prevalent TB was as strong as the association with HIV and independent from it, suggesting that in this setting household SEP and HIV may be equally important as determinants of prevalent TB. The strength of the association between HIV and prevalent TB we reported is similar to that reported in three other studies conducted in Zimbabwe and South Africa reporting measures of associations between 2.0 and 4.1 [42], [43], [44]. The lack of a stronger effect from HIV could be due to the fact that often the association of HIV with TB prevalent cases is generally lower than that observed for incident TB, for which measures of association typically range between 6 and 10 [45].

The existence of a strong, independent effect of household SEP should not undermine the importance of HIV in fuelling the TB epidemic in Zambia, rather it suggests that the epidemiological context in which the TB epidemic started and it is still maintained is likely to be complex. TB rates in Zambia dramatically increased only when the HIV epidemic started (end of 1980s'). However, data from the World Bank [46] suggest that early 90's were also years characterised by the worst contraction of the Zambian GDP, falling by 11% in 1994 and a further 5% in 1995 as a result of economy liberalisation and the end to most of the government subsidies for agriculture. During the period 1991–1998 poverty increased in the urban areas, as a consequence of the reduction in the employment in the parastatal sector. Furthermore, between 2001 and 2003 Zambia experienced its last major drought. This resulted into a 29% decline of the cereal production in year 2000–2001 compared to the previous year and a further decline in the following crop season due to more extended drought that affected larger parts of the country [46]. At the same time, maize prices increased up to 5 times higher than the five-year average and in some regions of the country maize was not available on the market [46]. Furthermore, the dietary energy supply in Zambia (Kcal/day per person) dropped in the past several decades and index of domestic food production has not shown any sign of increase between 1990 and 2000 [46]. Apparently these events did not affect the levels of acute malnutrition in young children (which stayed far below the level observed in case of famine or ongoing conflict), but the Zambian DHS documents a constant increase of chronic adult malnutrition over the 1990s [46].

All the above evidence may suggest that over this period TB transmission may have been driven and maintained by two forces: a high burden of household poverty and a rapidly developing HIV epidemic.

### The mechanism underlying the association between household SEP and prevalent TB

In this study the association between household SEP and prevalent TB was largely captured by the food intake-related variables to the extent that almost no effect of household SEP was left once the effect of food availability was taken into account. Thus, inadequate food intake might, be considered as the ‘*active ingredient*’ of the association under study [41]. The identification of a mediating factor is important because it supports the biological plausibility of the association under study, thus providing evidence that this association may be causal [41]. This plausibility is supported by several ecological studies and under different animal models and human data suggesting the key role of animal protein deprivation in TB infection and development [47], [48]. The importance of inadequate food-intake in the transmission of TB in these communities is further consistent with the secular data described above. Other nutrition-related factors could have been considered such as micronutrient intake and/or overall calories intake. However, there is little evidence that micronutrients deficiency plays a role in TB aetiology [49]. Furthermore, the collection of data on calories intake would have required the use of expensive and cumbersome food diaries that are unsuitable for the cross-sectional nature of this study.

### The other risk factors for prevalent TB

The protective effect of BCG was somewhat surprising given the extremely variable efficacy of BCG (0–80%) demonstrated in clinical trials [50]. Because of the very small number of subjects lacking BCG vaccination these results should be interpreted with caution, especially considering that the attribution of BCG status was made simply by scar reading and it was therefore prone to considerable observer variation [51].

The independent association with migration was also somewhat unexpected. Demographic data suggest that internal migration has become the predominant migration pattern for Zambia with a marked increase for migration towards less urban and supposedly less economically developed areas [52]. Although surprising, this could reflect the employment's reduction in the parastatal sector we mentioned before and therefore the lower household SEP of the people migrating. However, in this study we observed no confounding effect from the household SEP. Given the higher exposure to HIV among migratory workers [46], it also seems surprising the lack of any confounding effect of HIV on the effect of migration. It could be that people undergoing a migration experience may have been exposed to psychological stress and depression-related symptoms which ultimately may have increased their vulnerability to TB. This is plausible [53], but it cannot be confirmed with the available data.

### Preventing TB

In this study respectively 36% and 42% of prevalent cases could be attributed to HIV and inadequate food intake, respectively. These figures are consistent with what observed in other studies [43], [54], although their interpretation must be cautious because of the many, unverifiable assumptions required to compute them. If the associations we have identified actually reflected a causal relation between both nutrition and HIV with TB, then changing the distribution of these variables in the population would have the potential to prevent prevalent TB. It could be argued, for example, that by removing HIV from this population 36% of the current cases of prevalent TB may. More importantly, these figures would imply that raising the weekly number of meals containing proteins in each household to twice/week or more could reduce the burden of TB as much as removing HIV from this population. This of course doesn't mean that these interventions are equally doable, but efforts on both fronts would probably strengthen TB control, especially in contexts like this one where people are likely to experience both food insecurity and HIV infection at some point in their life.

The calculated PAFs also identify migration as an important to contributor to the case load in this population. Migration is likely not to be an actual cause for the disease, but rather a proxy for risk factors that are not captured by HIV infection and poor nutritional status.

### Conclusions

The evidence presented in this study suggests that today in Zambia socioeconomic factors are as important in TB epidemiology as they used to be 100 years ago in Europe and North America. The emergence of HIV has undoubtedly posed unprecedented challenges to the fight against TB and as a result a large proportion of TB research over the past two decades has been rightly invested to address this dual-epidemic. However, HIV seems to have not diminished the role of socioeconomic factors, particularly high-quality food availability. Although no definitive conclusion can be drawn, this evidence collectively seem to suggest that the fight against TB in Zambia could benefit from a broader approach moving beyond the HIV-TB coordinated efforts and incorporating interventions addressing household SEP. While few would argue with this suggestion, further research is necessary to understand which intervention should be prioritised, how to turn this aspiration into concrete actions, and what would be the impact and the cost-effectiveness of a similar approach. Given the threats posed by the current global food and financial crisis, investments in studies addressing these research questions both in developing and industrialised countries have never been more needed.
